# Associations between physical exercise and mental health among middle school students: potential psychosocial pathways and contextual heterogeneity from a healthy China perspective

**DOI:** 10.3389/fpsyg.2026.1831018

**Published:** 2026-07-01

**Authors:** Juanhui Zhang, Yan Liu

**Affiliations:** Department of Physical Education, Xinjiang University of Finance and Economics, Urumqi, China

**Keywords:** academic stress, family economic status, mental health, middle school students, peer context, physical exercise, school sports facilities

## Abstract

**Introduction:**

Physical exercise has been increasingly linked to adolescent mental health, but less is known about the psychosocial pathways and contextual conditions underlying this association. This study examines the relationship between physical exercise and mental health among Chinese middle school students, focusing on academic stress, peer context, school sports facilities, and family economic status.

**Methods:**

Using data from the China Education Panel Survey, this study analyzed a final sample of 6,713 middle school students. Fixed-effects models were used to examine the association between exercise duration and mental health, controlling for individual, family, school, and city-level factors. Robustness checks, instrumental-variable analysis, mediation analysis, and heterogeneity analysis were further conducted.

**Results:**

Longer physical exercise duration was positively associated with better mental health. This association remained stable across alternative specifications. Mediation analyses suggested that academic stress and peer context may serve as potential psychosocial pathways. Specifically, longer exercise duration was associated with lower academic stress and more favorable peer-related contexts, which were in turn associated with better mental health. Heterogeneity analyses showed that the positive association was stronger among students attending schools with sports facilities and those from families with higher economic status.

**Discussion:**

These findings provide large-sample observational evidence on the association between physical exercise and adolescent mental health in China. They suggest that school-based physical activity may be linked to mental health through academic and peer context, while its benefits may vary across school and family resources. Given the observational and cross-sectional nature of the data, the findings should be interpreted as associations rather than causal effects.

## 1 Introduction

Adolescent mental health has become a central concern in psychology, education, and public health. Middle school students are in a transitional developmental stage characterized by rapid changes in identity formation, emotional regulation, academic adaptation, and peer relationships (Steinberg, 2005; Blakemore and Mills, 2014; Degges-White, 2017). During this period, psychological distress may not only impair students' current learning and social adjustment but also have long-term consequences for wellbeing and human capital development (Hoover and Bostic, 2021; Kiessling and Norris, 2023). Therefore, identifying accessible and school-based behavioral factors associated with adolescent mental health remains an important research question.

Physical exercise represents one such factor because it is embedded in students' daily school routines and can be implemented more broadly than clinical or highly individualized interventions. Prior studies suggest that physical activity is associated with lower levels of anxiety and depressive symptoms, better self-related perceptions, and stronger social connectedness among adolescents (Biddle and Asare, 2011; Liu et al., 2015; Rodriguez-Ayllon et al., 2019). Compared with individualized interventions, school-based physical activity is relatively scalable and closely connected with students' educational environments (Eime et al., 2013; Lubans et al., 2016). However, the association between physical exercise and adolescent mental health is unlikely to be purely direct. In school settings, this association may be related to academic stress and peer context, especially as students face academic demands and frequent peer interactions during middle school (Fitzgerald et al., 2012; Lubans et al., 2016).

In the Chinese context, this issue is particularly relevant. The “Healthy China 2030” strategy and recent policy initiatives on student mental health have emphasized the importance of integrating physical health, psychological development, school education, and family support (Bai et al., 2022; Zhao et al., 2023; Yuan et al., 2024). These policy efforts provide an important institutional background for promoting adolescent wellbeing. Nevertheless, from an academic perspective, more empirical evidence is needed to clarify whether physical exercise is associated with better mental health among middle school students, through which mechanisms this relationship occurs, and whether the benefits of exercise are equally distributed across different school and family environments (Song, 2024).

Although prior research has documented a positive association between physical activity and mental health, several limitations remain. First, many studies rely on small-scale surveys or specific regional samples, which may limit the generalizability of their findings. Second, existing studies often focus on the direct association between exercise and mental health, while paying less attention to the psychosocial mechanisms linking the two (Lubans et al., 2016; Rodriguez-Ayllon et al., 2019). For middle school students, academic stress and peer relationships are two particularly important channels. Exercise may help students relieve academic stress, but it may also influence mental health by reshaping peer interaction, social belonging, and exposure to positive or negative peer behaviors (Pascoe et al., 2020; De La Haye et al., 2011; Fitzgerald et al., 2012). Third, the mental health benefits of exercise may vary across structural conditions. Students from families with different socioeconomic status or schools with different levels of sports facilities may not have equal access to high-quality physical activity opportunities, which may lead to heterogeneous effects (Ferreira et al., 2007; Stalsberg and Pedersen, 2010; Hanson and Chen, 2007).

To address these gaps, this study uses data from the China Education Panel Survey to examine the relationship between physical exercise and the mental health of middle school students. Specifically, we investigate whether longer exercise duration is associated with better mental health, whether academic stress and peer context serve as mediating pathways, and whether the association differs by school sports facilities and family economic status. By incorporating individual, family, school, and city-level factors into the empirical framework, this study provides a more comprehensive understanding of how physical exercise relates to adolescent mental health in the Chinese educational context.

This study contributes to the literature in three ways. First, it provides large-sample evidence on the association between physical exercise and middle school students' mental health using nationally representative CEPS data. Second, it moves beyond the direct-effect framework by examining academic stress and peer context as two psychosocial mechanisms. Third, it highlights the role of structural conditions by showing how school sports facilities and family economic status may shape the mental health benefits of physical exercise. These findings help clarify not only whether physical exercise matters for adolescent mental health, but also how and for whom it may be more effective.

## 2 Literature review and research hypotheses

### 2.1 Physical exercise and adolescent mental health

Existing research has provided substantial evidence on the relationship between physical activity and adolescent mental health. Early and recent studies have shown that regular physical activity is associated with lower levels of depression, anxiety, and psychological distress, as well as higher levels of self-esteem, emotional regulation, and life satisfaction among adolescents (Stephens, 1988; Biddle and Asare, 2011; Rodriguez-Ayllon et al., 2019; Maugeri et al., 2020; Wang et al., 2023). More recent work has further extended this literature by placing physical activity within a broader movement-behavior framework. From this perspective, adolescents' psychological functioning is not only related to exercise participation, but also to sedentary behavior and 24-h movement patterns (Gao et al., 2024; Zou et al., 2024; Zhang et al., 2025a). Nevertheless, the existing literature still provides limited evidence on how physical exercise is associated with mental health among Chinese middle school students, through which school-related psychosocial factors this association emerges, and whether it differs across family and school resource conditions.

Theoretically, physical exercise may affect mental health through both physiological and psychosocial mechanisms. From a physiological perspective, exercise can stimulate neurobiological processes related to endorphins, serotonin, dopamine, and stress regulation, which may reduce negative emotions and improve mood stability (Wegner et al., 2014; McEwen, 2007). From a psychosocial perspective, participation in exercise can enhance self-efficacy, perceived competence, social interaction, and interpersonal support (Fraser-Thomas et al., 2005; Lubans et al., 2016; Eime et al., 2013). These mechanisms suggest that physical exercise is not merely a health behavior but also an important developmental activity embedded in adolescents' daily school and social life. Mechanism-oriented reviews have further emphasized that physical activity may be linked to youth cognitive and mental health through interacting neurobiological, psychosocial, and behavioral processes (Lubans et al., 2016).

However, previous studies have several limitations. First, many studies focus on the general association between exercise and mental health without sufficiently examining the intermediate mechanisms through which this association occurs. Second, much of the existing evidence is based on small-scale or regional samples, making it difficult to generalize the findings to broader student populations. Third, relatively limited attention has been paid to whether the mental health benefits of exercise differ across family and school contexts (Ferreira et al., 2007; Stalsberg and Pedersen, 2010). These limitations suggest the need for a more integrated framework that examines not only whether physical exercise is associated with better mental health, but also how and under what conditions this relationship emerges.

### 2.2 Psychosocial pathways: academic stress and peer context

Physical exercise may influence adolescent mental health through multiple psychosocial pathways. In the school context, academic stress and peer relationships are two particularly important mechanisms. Middle school students often face high academic demands, examination pressure, and parental expectations (Sun et al., 2011). These pressures may increase anxiety, fatigue, frustration, and depressive symptoms (Liu and Lu, 2012; Pascoe et al., 2020; Steare et al., 2023). Physical exercise may help reduce academic stress by providing emotional release, improving stress regulation, and creating a temporary separation from academic tasks (McEwen, 2007; Lubans et al., 2016). Recent studies on movement behaviors and academic-related outcomes also suggest that physical activity may be connected to academic engagement, academic achievement, and executive functioning among children and adolescents (Bao et al., 2024; Gao et al., 2024; Zhang et al., 2025b). Classroom-based evidence further indicates that brief physical exercise before class may be associated with improved mathematics-specific inhibitory control, highlighting the relevance of executive processes in school settings (Zhang et al., 2026). Therefore, exercise may contribute to better mental health partly by alleviating students' perceived academic stress.

At the same time, this relationship should not be interpreted in an overly deterministic way. Physical exercise does not necessarily reduce academic stress for all students or in all contexts. If exercise crowds out study time or is experienced as an additional obligation, it may not produce the same psychological benefits. Therefore, academic stress should be understood as a possible mediating pathway rather than a universal causal channel.

Peer relationships represent another important psychosocial pathway. Adolescents spend substantial time with classmates and friends, and peer interaction plays a key role in emotional adjustment, social identity, and psychological wellbeing (Thoits, 2011; Pachucki et al., 2015; Oldfield et al., 2018; Luijten et al., 2023). In this study, peer context refers to students' perceived exposure to peers' behaviors and social interactions. Physical exercise, especially in school settings, often involves teamwork, cooperation, competition, and informal communication. These activities may create opportunities for students to build friendships, develop social support, and experience a sense of group belonging (Fitzgerald et al., 2012; De La Haye et al., 2011; Eime et al., 2013). This interpretation is also consistent with evidence that physical activity programs may support social and emotional wellbeing among at-risk youth, particularly when they provide structured opportunities for interpersonal interaction and social support (Lubans et al., 2012).

However, peer context is not necessarily positive. While supportive peers may enhance confidence, belonging, and emotional security, negative peer behaviors may expose students to conflict, exclusion, risk-taking, or disciplinary problems (Gifford-Smith et al., 2005; Dishion and Tipsord, 2011). Therefore, the peer-related mechanism may have a dual nature: positive peer context may improve mental health, whereas negative peer context may weaken or offset the benefits of physical exercise. This distinction is important because it prevents the analysis from treating peer interaction as uniformly beneficial.

### 2.3 Contextual heterogeneity: school sports facilities and family economic status

The relationship between physical exercise and mental health may also depend on the contexts in which exercise takes place. For middle school students, school and family are two major environments shaping access to physical activity, the quality of exercise participation, and the psychological returns from exercise (Ferreira et al., 2007; Deforche et al., 2010).

School sports facilities are an important institutional condition for student exercise. Schools with adequate sports fields, gymnasiums, or other physical activity spaces can provide students with safer, more convenient, and more diverse opportunities for exercise. Better facilities may also increase the likelihood that physical activity is organized, regular, and socially supportive. By contrast, in schools with limited sports facilities, students may face higher participation costs, fewer activity options, and weaker institutional support (Ferreira et al., 2007; Deforche et al., 2010). Therefore, the mental health benefits of physical exercise may be stronger in schools with better sports facilities.

Family economic status is another important contextual factor. Students from higher economic-status families may have greater access to sports equipment, extracurricular training, health-related knowledge, and parental support for exercise participation. These resources may enhance the quality and continuity of physical activity, thereby strengthening its psychological benefits. In contrast, students from lower economic-status families may face resource constraints that limit their ability to transform exercise participation into sustained mental health improvement (Hanson and Chen, 2007; Stalsberg and Pedersen, 2010; Reiss, 2013). Thus, family economic status may condition the association between physical exercise and mental health.

This contextual perspective does not imply that exercise is unimportant for disadvantaged students. Rather, it suggests that unequal access to supportive family and school resources may shape the extent to which students benefit from exercise. Examining heterogeneity is therefore necessary for understanding whether physical exercise functions as an equalizing intervention or whether its benefits are constrained by structural inequalities (Xie et al., 2022; Li et al., 2025).

### 2.4 The hypotheses of this study

In summary, this study aims to examine the association between physical exercise and the mental health of Chinese middle school students. Building on the above theoretical discussion, we further explore whether this association operates through two psychosocial pathways—academic stress and peer context—and whether it varies across different school and family contexts. Specifically, we focus on school sports facilities and family economic status as two contextual conditions that may shape the mental health benefits of physical exercise.

**Figure 1:**
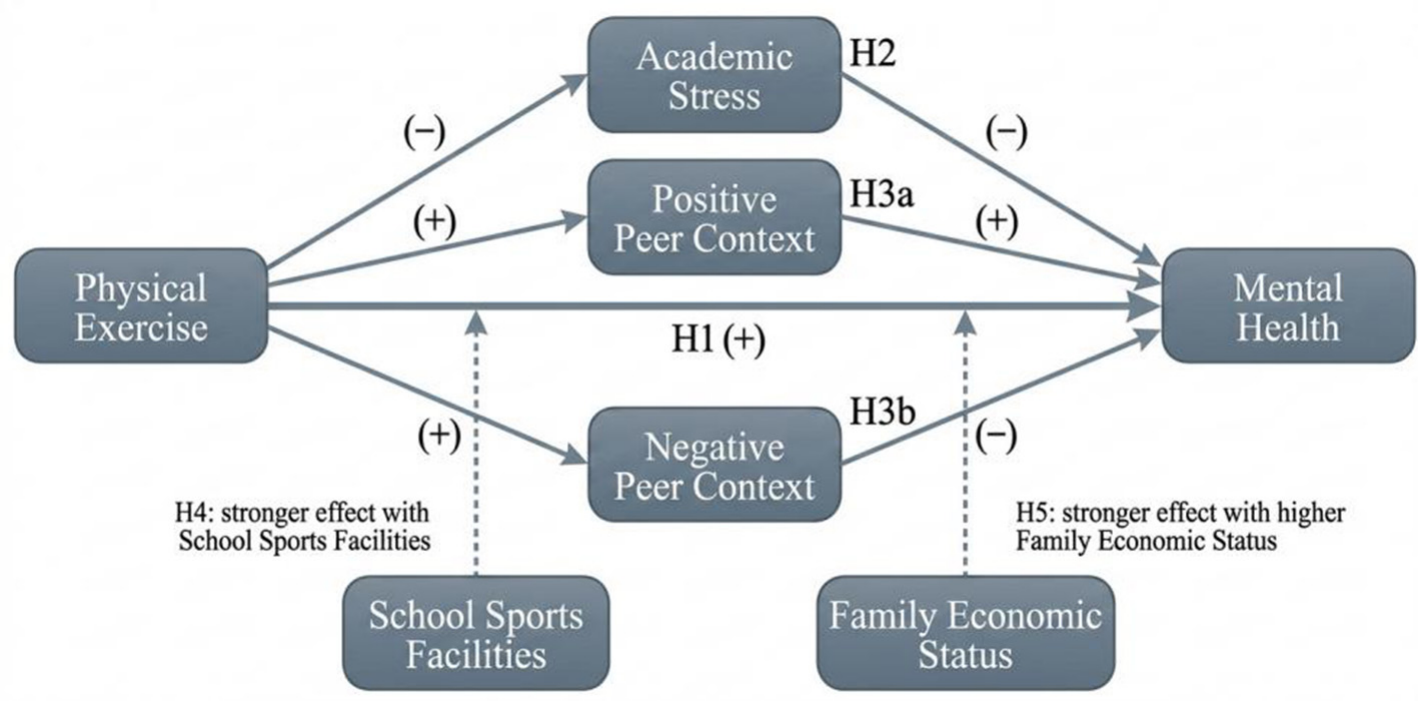
Conceptual framework of the relationship between physical exercise and adolescent mental health.

To present these relationships more clearly, Figure 1 provides the conceptual framework of this study. The framework shows that physical exercise may be directly associated with students' mental health and may also be indirectly associated with mental health through academic stress, positive peer context, and negative peer context. In addition, school sports facilities and family economic status are incorporated as contextual factors that may condition the strength of the association between physical exercise and mental health. Based on this conceptual framework and the preceding literature review, the following hypotheses are proposed:

H1: Physical exercise is positively associated with the mental health of middle school students.
H2: Academic stress mediates the association between physical exercise and mental health. Specifically, physical exercise is associated with lower academic stress, which in turn is associated with better mental health.
H3a: Positive peer context mediates the association between physical exercise and mental health. Specifically, physical exercise is associated with more favorable peer contexts, which in turn are associated with better mental health.
H3b: Negative peer context mediates the association between physical exercise and mental health in the opposite direction. Specifically, physical exercise may be associated with greater exposure to negative peer contexts, which in turn are associated with poorer mental health.
H4: The positive association between physical exercise and mental health is stronger among students attending schools with sports facilities.
H5: The positive association between physical exercise and mental health is stronger among students from families with higher economic status.

## 3 Research design

### 3.1 Data source

The data for this study were sourced from the China Education Panel Survey (CEPS), designed and implemented by the China Survey and Data Center at Renmin University of China. The survey targets middle school students, their parents, homeroom teachers, subject teachers, and school administrators, providing nationally representative data.

Specifically, this study used the CEPS 2014–2015 follow-up data and focused on Grade 8 students. This choice follows the official CEPS design: although the baseline survey included both Grade 7 and Grade 9 cohorts, the Grade 9 cohort was an experimental sample, while the 2014–2015 follow-up formally tracked students who were in Grade 7 at baseline and had progressed to Grade 8. The follow-up survey successfully tracked 9,449 of the 10,279 baseline Grade 7 students, with a follow-up rate of 91.9%, ensuring a reliable analytical sample. Therefore, using Grade 8 students and their corresponding parent questionnaires is consistent with the CEPS panel design and helps link student, family, and school-level information. This study utilized three CEPS datasets: the Grade 8 Student Questionnaire, the corresponding Parent Questionnaire, and the School Administrator Questionnaire. These datasets were merged using the official student and school identifiers provided by CEPS.

Of the 9,906 original observations, 3,193 observations, accounting for approximately 32.2% of the original sample, were excluded because of missing values in the variables used in the empirical analysis. After listwise deletion, 6,713 valid observations were retained for the complete-case analysis. We further examined the distribution of missing values across the variables used in the merged dataset. The results showed that missingness was distributed across multiple student-, family-, school-, health-, and behavior-related variables rather than being driven by a single variable or a single category of variables. Although several family-background items showed somewhat higher missing proportions than other variables, the highest missing proportion for any single variable was only approximately 5%. This pattern suggests that missingness was broadly dispersed across the dataset rather than concentrated in a narrow set of family socioeconomic indicators.

The missing values may have arisen from several sources, including incomplete questionnaire responses, item-level non-response, respondents' limited knowledge of detailed family-background information, and the relatively sensitive nature of some parental-background questions such as political affiliation. Because the excluded observations accounted for a non-negligible proportion of the original sample and missingness was distributed across multiple variables, we do not assume that the data are strictly Missing Completely at Random. To provide additional evidence on the potential implications of missingness, we compared core study variables between retained and excluded observations. The two groups showed negligible differences in mental health (Cohen's *d* = −0.048) and physical exercise (Cohen's *d* = −0.081), suggesting that missingness was not substantively associated with the primary outcome or the key explanatory variable in the observed data. In addition, supplementary analyses using Multiple Imputation by Chained Equations with 20 imputed datasets yielded substantively consistent conclusions. These results suggest that the main findings are unlikely to be driven solely by the use of listwise deletion, although we continue to interpret the estimates as conditional associations rather than definitive causal effects.

### 3.2 Variable selection

#### 3.2.1 Dependent variable

The dependent variable is adolescent mental health, measured using students' responses to 10 items asking whether they had experienced specific feelings in the past 7 days. These items include depression, inability to concentrate, unhappiness, feelings of meaninglessness, lack of motivation, sadness, nervousness, excessive worry, premonition of bad events, and over-excitability leading to inattention in class. Following previous CEPS-based studies, the original response categories were reverse-coded so that higher values indicate better mental health (Yu et al., 2020). The average score of the 10 items was then multiplied by 100 to construct a continuous mental health index ranging from 0 to 100. The detailed coding procedure is reported in Table 1.

**Table 1:** Variable definitions and coding.

Variable	Variable type	Measurement	Coding
Mental health	Dependent variable	Average score of 10 mental health items from the student questionnaire	Original responses were reverse-coded so that higher values indicate better mental health; the average score was multiplied by 100
Physical exercise	Key independent variable	Weekly physical exercise duration	Exercise days per week × minutes per exercise day; divided by 100 in regression
Academic stress	Mediating variable	Constructed from academic course stress and parental educational expectations	PCA-based index; higher values indicate greater academic stress
Positive peer context	Mediating variable	Friends' positive behaviors, including excellent academic performance, diligent study habits, and aspirations to attend university	Each item coded as 0 = no such situation, 1 = one or two friends, 2 = many friends; item scores summed
Negative peer context	Mediating variable	Friends' negative behaviors, including skipping class, rule violation, fighting, smoking/drinking, internet cafe or gaming hall visits, early romantic relationships, and dropping out of school	Each item coded as 0 = no such situation, 1 = one or two friends, 2 = many friends; item scores summed
Gender	Individual control	Student gender	0 = male; 1 = female
Only child	Individual control	Whether the student is an only child	0 = non-only child; 1 = only child
Household registration type	Individual control	Student household registration status	0 = non-agricultural; 1 = agricultural; 2 = resident; 3 = no registration
Boarding student	Individual control	Whether the student boards at school	0 = non-boarding; 1 = boarding
Physical health	Individual control	Student self-rated physical health	0 = very poor; 1 = poor; 2 = fair; 3 = good; 4 = very good
Parental divorce	Family control	Whether parents are divorced	0 = yes; 1 = no
Relationship with parents	Family control	Quality of parent-child relationship	0 = poor; 1 = good
Parental education level	Family control	Average educational attainment of both parents	Averaged across father's and mother's education levels
Parental occupation	Family control	Average of both parents' occupational categories	High-skill occupation = 1; other occupations = 0; averaged across parents
Household economic status	Heterogeneity variable	Parent-reported household economic condition	Low status: responses 1–2; non-low status: responses 3–5
Teacher-student relationship	School control	Student-reported relationship with homeroom and subject teachers	Average score; higher values indicate better teacher-student relationship
School ranking	School control	Principal's assessment of school performance within the county/district	Recoded from 0 = worst to 4 = best
School type	School control	Type of school	0 = public; 1 = migrant private; 2 = general private; 3 = subsidized private
Non-sports facilities	School control	Availability of non-sports facilities, including libraries, activity rooms, cafeterias, counseling rooms, music rooms, laboratories, and computer rooms	Average facility index
School sports facilities	Heterogeneity variable	Whether the school has a swimming pool, sports field, or gymnasium	0 = no sports facilities; 1 = at least one sports facility

#### 3.2.2 Key independent variable

The key independent variable is students' weekly physical exercise duration. Following the common frequency-duration approach used in survey-based physical activity measurement, this variable was constructed from two CEPS questions: “How many days a week do you usually exercise?” and “How many minutes do you exercise per day?” For each student, weekly exercise duration was calculated by multiplying the number of exercise days per week by the average minutes of exercise per day. The resulting value represents total minutes of physical exercise per week. To facilitate coefficient interpretation, the weekly exercise duration was divided by 100 in the regression analysis. Observations reporting more than 360 min of exercise in a single day were treated as implausible outliers and excluded from the analysis. The detailed construction and coding are shown in Table 1.

#### 3.2.3 Control variables

Control variables were categorized into individual, family, and school levels. To improve consistency and replicability, all categorical variables were coded using a unified format. Binary variables were coded as 0 for the reference category and 1 for the target category. Ordinal variables were coded in ascending order, with higher values indicating a higher level of the corresponding attribute. Composite variables were constructed by summing or averaging the relevant items, as described in Table 1.

At the individual level, the control variables include gender, only-child status, household registration type, boarding status, and self-rated physical health. These variables capture basic demographic characteristics and health conditions that may be associated with students' mental health.

At the family level, the control variables include parental divorce status, parent-child relationship, parental education level, and parental occupation. Parental education level was calculated as the average educational attainment of both parents, and parental occupation was constructed by first classifying each parent's occupation as high-skill or other occupations and then averaging the two parental occupation indicators (Pachucki et al., 2015). Household economic status was used as a heterogeneity variable. It was obtained from the parent questionnaire item asking respondents to evaluate their current household economic condition. Responses indicating “very difficult” or “fairly difficult” were classified as the low household economic status group, while responses indicating “average,” “fairly wealthy,” or “very wealthy” were classified as the non-low household economic status group.

At the school level, the control variables include teacher-student relationship, school ranking, school type, and non-sports facilities. Teacher-student relationship was measured using students' reported relationship with homeroom and subject teachers. School ranking was derived from the School Administrator Questionnaire and reflects the principal's assessment of the school's relative performance within the county or district. School type and non-sports facilities were also obtained from the school-level questionnaire. The detailed variable definitions and coding procedures are reported in Table 1.

#### 3.2.4 Mediator variables

Both mediator variables were derived from the 8th Grade Student Questionnaire of the China Education Panel Survey for the 2014–2015 academic year. Academic stress captures students' perceived learning burden and educational pressure, while peer context reflects students' perceived peer environment based on their friends' behaviors. To maintain consistency in variable construction, the detailed coding procedures for both mediator variables are summarized in Table 1.

##### 3.2.4.1 Academic stress

Academic stress was constructed from students' perceived academic course stress and parental educational expectations. Academic course stress was measured using students' reported difficulty in learning Chinese, mathematics, and English. Parental educational expectations were measured using students' reports of their parents' expected educational attainment for them. Reliability and validity tests indicated that these items were suitable for index construction. Specifically, Cronbach's alpha was 0.86, suggesting good internal consistency. Principal component analysis was then performed, yielding a Kaiser-Meyer-Olkin statistic of 0.644 and a Bartlett's test of sphericity significant at the 0.01 level. These results indicate that the data are appropriate for principal component analysis. The resulting index was used to measure academic stress, with higher values indicating greater academic stress.

##### 3.2.4.2 Peer context

In this study, peer context refers to students' perceived peer environment, measured by the behaviors exhibited by their friends. We use the term “peer context” rather than “peer effects” to avoid implying strict causal peer influence. Drawing on prior research on peer context and adolescent development (Kiessling and Norris, 2023; Tang et al., 2025), two variables were constructed: positive peer context and negative peer context.

Positive peer context was constructed from students' reports of whether their friends displayed positive behaviors, including excellent academic performance, diligent study habits, and aspirations to attend university. Each item was coded using the same three-category format: 0 = no such situation, 1 = one or two friends exhibit this behavior, and 2 = many friends exhibit this behavior. The item scores were summed to construct the positive peer context variable, with higher values indicating a more positive perceived peer environment.

Negative peer context was constructed from students' reports of whether their friends displayed negative behaviors, including skipping class, being absent without permission, violating school rules, receiving disciplinary actions, fighting, smoking or drinking, frequently visiting internet cafes or gaming halls, early romantic relationships, and dropping out of school. Each item was coded using the same three-category format: 0 = no such situation, 1 = one or two friends exhibit this behavior, and 2 = many friends exhibit this behavior. The item scores were summed to construct the negative peer context variable, with higher values indicating greater exposure to negative peer behaviors.

### 3.3 Model construction

Given the hierarchical structure of the CEPS data, students are nested within schools and city-level sampling units. Therefore, in model construction, this study controls for student-, family-, and school-level characteristics to reduce potential omitted-variable bias. In addition, the CEPS data provide anonymized school and city identifiers, which allow us to account for contextual heterogeneity across schools and cities to the extent permitted by the dataset. According to the CEPS data manual, the baseline survey randomly selected 28 county-level units and 112 schools, and all students in the selected classes were included in the survey.

It is important to note that, in adherence to privacy and data protection policies, CEPS does not disclose the actual geographic identifiers corresponding to city and school IDs. According to the CEPS research team, revealing the names of provinces, cities, or districts could lead to regional ranking or comparison and potentially result in misuse of the data. As such, researchers only have access to anonymized identifiers for cities and schools and are unable to link them to specific geographic locations.

Due to these data restrictions, we are unable to include concrete city-level covariates in the model, such as GDP per capita, educational expenditure, or urbanization rate. To account for unobserved contextual differences, this study includes both school fixed effects and city fixed effects. Specifically, school fixed effects control for time-invariant differences across schools, such as school climate, management practices, and general educational environment, while city fixed effects capture broader contextual differences across anonymized city-level sampling units. This fixed-effects specification is consistent with previous CEPS-based studies that use available administrative identifiers to account for multilevel contextual heterogeneity (Chang et al., 2023). However, because the data are observational and measured within the same survey wave, this specification should be understood as a way to reduce potential confounding rather than as a basis for strong causal identification.

It should be noted that the CEPS data used in this study are observational, and the key variables are measured within the same survey wave. Therefore, the empirical results should be interpreted as conditional associations rather than causal effects. The fixed-effects specification and control variables help reduce potential confounding from observed covariates and school- and city-level contextual heterogeneity, but they do not establish temporal ordering or definitive causal relationships among the key variables. Given the hierarchical structure of the data and the possibility that students within the same school share similar educational environments, standard errors are clustered at the school level. The baseline model is specified as follows:

$$\mathrm{Menta}\_\mathrm{health}h_{i}=\beta_{0}+\beta_{1}\mathrm{Exercise}e_{i}+\sum_{k=2}^{13}\beta_{k}\mathrm{Controls}s_{k,i}+\delta_{s}+\gamma_{c}+\epsilon_{i,s,c}$$

Here, *i* represents the individual student, *s* denotes the school, and *c* refers to the city. The dependent variable _*Mental*_*health*_*i*_ is the student's mental health status, while the key independent variable, *Exercise*_*i*_, represents the student's average weekly physical exercise duration. *Controls*_*k*_ denotes a set of control variables, δ_*s*_ captures school fixed effects, γ_*c*_ represents city fixed effects, and ϵ_*i, s, c*_ is the random error term. We use STATA software for modeling research, and descriptive statistics for the main variables used in this study are presented in Table 2.

**Table 2:** Descriptive statistics.

Variable name	Obs	Mean	SD	Min	Median	Max
Mental health	6,713	74.802	16.412	25.000	75.000	100.000
Physical exercise	6,713	1.529	1.640	0.000	1.200	25.200
Academic stress	6,713	−0.003	0.479	−1.665	−0.062	2.929
Peer context	6,713	11.009	16.614	0.000	6.000	99.000
Gender	6,713	0.498	0.500	0.000	0.000	1.000
Only child	6,713	0.457	0.498	0.000	0.000	1.000
Household registration type	6,713	0.913	0.696	0.000	1.000	3.000
Boarding student	6,713	0.296	0.456	0.000	0.000	1.000
Physical health	6,713	2.886	0.926	0.000	3.000	4.000
Parental divorce	6,713	0.927	0.260	0.000	1.000	1.000
Relationship with parents	6,713	0.901	0.298	0.000	1.000	1.000
Parental education level	6,713	3.174	1.849	0.000	2.500	8.000
Parental occupation	6,713	0.176	0.381	0.000	0.000	1.000
Teacher-student relationship	6,713	2.252	0.588	0.000	2.000	3.000
School ranking	6,713	3.020	0.832	0.000	3.000	4.000
School type	6,713	0.111	0.458	0.000	0.000	3.000
Non-sports facilities at school	6,713	1.352	0.503	0.143	1.571	2.000

## 4 Empirical results analysis

### 4.1 Baseline regression

This study employs the regression model specified in the baseline model to examine the relationship between physical exercise and the mental health of middle school students. The regression results are presented in Table 3. Column (1) reports the result when only physical exercise is included as the explanatory variable. Columns (2) and (3) further incorporate school fixed effects, city fixed effects, and control variables. Across different model specifications, the coefficient of physical exercise remains positive and statistically significant. Although the coefficient decreases after fixed effects and control variables are added, its sign and significance remain stable, suggesting a robust positive association between physical exercise duration and students' mental health. These findings support Hypothesis 1 of this study.

**Table 3:** Baseline regression results.

Variables	(1)	(2)	(3)
	Mental health	Mental health	Mental health
Physical exercise	0.886^***^	0.606^***^	0.269^**^
	(7.28)	(4.81)	(2.15)
Gender			−1.958^***^
			(−4.97)
Only child			0.737
			(1.54)
Household registration type			−0.227
			(−0.72)
Boarding student			−0.506
			(−0.65)
Physical health			3.366^***^
			(15.74)
Parental divorce			0.009
			(0.01)
Relationship with parents			1.878^***^
			(2.66)
Parental education level			0.545^***^
			(3.64)
Parental occupation			0.749
			(1.25)
Teacher-student relationship			2.792^***^
			(7.95)
School ranking			1.387
			(0.25)
School type			−2.905
			(−0.52)
Non-sports facilities at school			0.252
			(0.02)
Constant	73.448^***^	71.840^***^	48.670
	(269.15)	(26.44)	(1.50)
City fixed effects	No	Yes	Yes
School fixed effects	No	Yes	Yes
Observations (*N*)	6,713	6,713	6,713
Adjusted *R^2^*	0.008	0.047	0.104

^***^, ^**^, and ^*^ indicates statistical significance at the 1%, 5%, and 10% levels, respectively.

Regarding control variables, factors such as gender, physical health status, parent-child relationship, and teacher-student relationship remain significant after including fixed effects, highlighting the importance of these variables in the model. Specifically, the regression coefficient for gender is −1.958, indicating that female students have significantly lower mental health levels than male students. The coefficient for physical health status is significantly positive, suggesting that better physical health can effectively reduce psychological problems. Additionally, the coefficients for parent-child relationship and teacher-student relationship are both positive and significant at the 1% level, demonstrating that healthy family and school relationships contribute to better mental health. Furthermore, higher parental education levels are associated with improved mental health among students. However, after controlling for school and city fixed effects, variables such as being an only child, household registration type, boarding status, and parental marital status no longer exhibit significant effects on mental health.

### 4.2 Robustness checks

To examine the robustness of the baseline results, this study conducts two additional checks. First, we replace the continuous measure of physical exercise with a theoretically grounded binary indicator based on international physical activity recommendations. Specifically, the World Health Organization recommends that children and adolescents aged 5–17 should engage in an average of at least 60 min of moderate-to-vigorous physical activity per day, equivalent to approximately 420 min per week. Accordingly, students whose reported weekly exercise duration reaches or exceeds 420 min are coded as 1, and those below this threshold are coded as 0. Since the CEPS questionnaire records exercise frequency and duration but does not directly measure exercise intensity, this indicator should be interpreted as whether students meet the recommended exercise-duration threshold rather than full compliance with the WHO moderate-to-vigorous physical activity standard (World Health Organization, 2020). Second, we replace the continuous mental health score with a binary mental health indicator and estimate the model using a Probit specification. Table 4 presents the robustness-check results. The coefficient of physical exercise remains positive and statistically significant across the alternative specifications, suggesting that the positive association between physical exercise and students' mental health is not driven by the baseline variable construction or model specification. These results provide further support for Hypothesis 1.

**Table 4:** Robustness checks.

Variables	(1)	(2)
	Mental health	Mental health dummy
Physical exercise dummy	0.733^*^	
	(1.76)	
Physical exercise		0.021^**^
		(1.97)
Gender	−2.033^***^	−0.103^***^
	(−5.21)	(−3.14)
Only child	0.719	0.042
	(1.51)	(1.06)
Household registration type	−0.228	0.000
	(−0.73)	(0.02)
Boarding student	−0.497	−0.039
	(−0.64)	(−0.59)
Physical health	3.383^***^	0.223^***^
	(15.86)	(12.51)
Parental divorce	−0.013	0.001
	(−0.02)	(0.02)
Relationship with parents	1.865^***^	0.107^*^
	(2.64)	(1.81)
Parental education level	0.540^***^	0.033^***^
	(3.60)	(2.69)
Parental occupation	0.768	0.021
	(1.28)	(0.43)
Teacher-student relationship	2.759^***^	0.213^***^
	(7.85)	(7.28)
School ranking	1.126	0.054
	(0.20)	(0.12)
School type	−2.991	−0.356
	(−0.54)	(−0.77)
Non-sports facilities at school	0.298	−0.009
	(0.02)	(−0.01)
Constant	49.848	−1.688
	(1.53)	(−0.63)
City fixed effects	Yes	Yes
School fixed effects	Yes	Yes
Observations (*N*)	6,713	6,713
Adjusted *R^2^*	0.103	

^***^, ^**^, and ^*^ indicates statistical significance at the 1%, 5%, and 10% levels, respectively.

### 4.3 Endogeneity test

To further examine whether the baseline association is sensitive to potential endogeneity concerns, this study reports a two-stage least squares (2SLS) specification as a supplementary sensitivity analysis. Potential endogeneity may arise for two reasons. First, reverse association may exist between physical exercise and mental health, as adolescents' psychological conditions may also affect their willingness and ability to participate in physical exercise. For example, symptoms related to depression or anxiety may reduce students' participation in physical activity. Second, although this study controls for a series of individual-, family-, and school-level characteristics and includes school and city fixed effects, unobserved factors may still affect both physical exercise and mental health.

Following prior empirical research on physical exercise and adolescent mental health, this study uses school-level average physical exercise time as an instrumental variable for individual exercise time (Li et al., 2024). The rationale is that students' exercise behavior is partly shaped by the shared physical activity environment within schools, including general exercise atmosphere, physical education arrangements, and school-level exercise norms. Therefore, school-level average exercise time is expected to be closely related to individual students' exercise duration. At the same time, the model controls for individual-, family-, and school-level covariates, as well as school and city fixed effects, to reduce the influence of observed characteristics and fixed contextual differences.

The regression results are presented in Table 5. Columns (1) and (2) report the first-stage and second-stage regression results, respectively. The first-stage results show that the instrumental variable is positively and significantly associated with individual exercise time, indicating that the relevance condition is empirically supported. The under-identification test also rejects the null hypothesis of under-identification, with the Anderson LM statistic significant at the 0.000 level. In addition, the Cragg-Donald Wald F-statistic is 232.298, exceeding the Stock-Yogo critical value at the 10% level, suggesting that weak-instrument concerns are unlikely to dominate the estimation.

**Table 5:** Endogeneity test: two-stage least squares (2SLS).

Variables	(1)	(2)
	Physical exercise	Mental health
Average physical exercise time	0.962^***^	
	(15.24)	
Physical exercise		1.747^***^
		(2.58)
Gender	−0.500^***^	−1.267^**^
	(−13.20)	(−2.44)
Only child	−0.004	0.825^*^
	(−0.10)	(1.75)
Household registration type	0.033	−0.312
	(1.09)	(−1.01)
Boarding student	0.019	−0.789
	(0.31)	(−1.26)
Physical health	0.168^***^	3.150^***^
	(8.14)	(12.94)
Parental divorce	−0.020	−0.150
	(−0.26)	(−0.19)
Relationship with parents	−0.043	2.316^***^
	(−0.63)	(3.27)
Parental education level	0.026^*^	0.478^***^
	(1.86)	(3.21)
Parental occupation	0.023	0.567
	(0.39)	(0.94)
Teacher-student relationship	−0.002	2.890^***^
	(−0.07)	(8.35)
School ranking	−0.011	−0.675^**^
	(−0.38)	(−2.20)
School type	−0.029	−2.200^***^
	(−0.58)	(−4.34)
Non-sports facilities at school	−0.016	−0.036
	(−0.34)	(−0.07)
Constant	−0.156	53.423^***^
	(−0.54)	(18.35)
City fixed effects	No	No
School fixed effects	Yes	Yes
Observations (*N*)	6,713	6,713
Adjusted *R^2^*	0.135	0.078

^***^, ^**^, and ^*^ indicate statistical significance at the 1%, 5%, and 10% levels, respectively.

The second-stage results show that the coefficient of physical exercise remains positive and statistically significant. These findings are broadly consistent with the baseline regression results. However, because school-level average exercise time may also reflect broader school environments, the IV results should not be interpreted as definitive causal evidence. Rather, they are used as a supplementary sensitivity analysis to examine whether the positive association between physical exercise and adolescents' mental health remains stable under an alternative empirical specification. Overall, the 2SLS results provide additional support for the robustness of the baseline association.

### 4.4 Mediation analysis of potential psychosocial pathways

The previous analysis shows that physical exercise is significantly and positively associated with the mental health of middle school students. This association remains robust after replacing the key independent variable, changing the regression model, and addressing potential endogeneity concerns. However, given the observational and cross-sectional nature of the data, these findings should be interpreted as conditional associations rather than definitive causal effects.

From the perspective of school life, academic stress may represent an important psychosocial pathway linking physical exercise and mental health. On the one hand, academic performance is a primary concern for many parents and students. Increased extracurricular physical activity may reduce available study time and, under certain conditions, may be perceived as creating additional academic stress (Tang et al., 2025). On the other hand, students who engage in more frequent physical exercise may experience lower levels of academic stress, possibly because exercise provides emotional release, supports stress regulation, and creates temporary psychological separation from academic tasks. Recent studies have also emphasized that movement behaviors are closely related to academic-related outcomes, executive functioning, and psychological wellbeing among children and adolescents (Lubans et al., 2016; Bao et al., 2024; Zhang et al., 2026). Therefore, this study further examines whether academic stress is statistically associated with the relationship between physical exercise and mental health.

In addition, due to the dense peer interaction environment of schools, peer context may also serve as a potential pathway linking physical exercise and mental health. School-based physical exercise often involves teamwork, cooperation, competition, and informal communication, which may be associated with more favorable peer interactions and a stronger sense of belonging. At the same time, peer-related contexts may also include exposure to negative peer behaviors. Prior research on team sports and adolescent development has also suggested that physical activity may be linked to mental health through social and peer-related contexts (Easterlin et al., 2019). Therefore, this study distinguishes between academic stress and peer context as two potential psychosocial pathways. Following the methodology of (MacKinnon et al. 2000), a mediation effect model is constructed, where *media*_**i**_ represents the mediating variables: academic stress and peer context.

$$\begin{array}{l} Mental\_health_{i}=\beta_{0}+\beta_{1}\;exercise_{i}+\beta_{2}x_{i}+\varepsilon_{i}0\\ \;media_{i}=\mu_{0}+\mu_{1}\;exercise_{i}+\mu_{2}x_{i}+\varepsilon_{i}\\ Mental\_health_{i}=\gamma_{0}+\gamma_{1}\;exercise_{i}+\gamma_{2}\;media\;+\gamma_{3}x_{1}\varepsilon_{i} \end{array}$$ (1)

The mediation analysis results for “academic stress” are presented in Table 6. Column (2) of Table 6 shows that the regression coefficient of physical exercise on the mediating variable, academic stress, is significantly negative. Similarly, Column (3) demonstrates that the regression coefficient of academic stress on mental health is also significantly negative. These findings suggest a statistically significant indirect association through academic stress. Specifically, physical exercise duration is negatively associated with academic stress, and academic stress is negatively associated with mental health. This pattern indicates that students who report longer exercise duration tend to report lower academic stress, which is in turn associated with better mental health. Furthermore, robustness checks using the Sobel test and Bootstrap method confirm the reliability of these conclusions, providing strong support for Hypothesis 2.

**Table 6:** Mediation analysis: academic stress.

Variables	(1)	(2)	(3)
	Mental health	Academic stress	Mental health
Average physical exercise time	0.269^**^	−0.015^***^	0.209^*^
	(2.15)	(−2.92)	(1.69)
Physical exercise			−4.071^***^
			(−13.62)
Gender	−1.958^***^	−0.174^***^	−2.665^***^
	(−4.97)	(−10.87)	(−6.81)
Only child	0.737	−0.021	0.653
	(1.54)	(−1.06)	(1.39)
Household registration type	−0.227	0.052^***^	−0.016
	(−0.72)	(4.07)	(−0.05)
Boarding student	−0.506	0.036	−0.358
	(−0.65)	(1.14)	(−0.46)
Physical health	3.366^***^	−0.095^***^	2.980^***^
	(15.74)	(−10.91)	(14.01)
Parental divorce	0.009	−0.020	−0.071
	(0.01)	(−0.60)	(−0.09)
Relationship with parents	1.878^***^	−0.128^***^	1.356^*^
	(2.66)	(−4.48)	(1.95)
Parental education level	0.545^***^	−0.065^***^	0.282^*^
	(3.64)	(−10.60)	(1.89)
Parental occupation	0.749	−0.027	0.638
	(1.25)	(−1.12)	(1.08)
Teacher-student relationship	2.792^***^	−0.193^***^	2.007^***^
	(7.95)	(−13.52)	(5.72)
School ranking	1.387	0.093	1.766
	(0.25)	(0.41)	(0.32)
School type	−2.905	0.035	−2.762
	(−0.52)	(0.15)	(−0.50)
Non-sports facilities at school	0.252	0.403	1.893
	(0.02)	(0.71)	(0.14)
Constant	48.670	0.233	49.617
	(1.50)	(0.18)	(1.55)
Sobel test	Mediator variable: Academic stress −3.868^***^ Indirect association—Negative conduction		
Ind_eff Test (P-val)	0.017 Indirect effect established		
City fixed effects	Yes	Yes	Yes
School fixed effects	Yes	Yes	Yes
Observations (*N*)	6,713	6,713	6,713
Adjusted *R^2^*	0.104	0.220	0.128

^***^, ^**^, and ^*^ indicate statistical significance at the 1%, 5%, and 10% levels, respectively.

The model outputs indicate that physical exercise duration is significantly associated with peer context, suggesting that middle school students who engage in longer exercise duration are more likely to report friendships and a sense of group belonging during physical activities. After including both the key independent variable and the mediating variable in the model, the coefficients for both peer context and physical exercise remain significantly positive. This supports the mediating role of peer context in the relationship between physical exercise and mental health, indicating that longer physical exercise duration may be linked to better adolescent mental health through more favorable peer context, thereby supporting Hypothesis 3. Detailed results are presented in Columns (1) to (3) of Table 7. Robustness checks using the Sobel test and Bootstrap method further confirm the stability of the mediating effect of peer context.

**Table 7:** Mediation analysis: peer context.

Variables	(1)	(2)	(3)	(4)	(5)	(6)	(7)
	Mental health	Peer context	Mental health	Positive peer context	Mental health	Negative peer context	Mental health
Physical exercise	0.269^**^	0.708^***^	0.240^*^	0.021^***^	0.236^*^	0.081^***^	0.318^**^
	(2.15)	(5.49)	(1.92)	(4.52)	(1.89)	(5.02)	(2.55)
Peer context			0.041^***^				
			(3.40)				
Positive peer context					1.599^***^		
					(4.73)		
Negative peer context							−0.605^***^
							(−6.36)
Constant	48.670	−5.376	48.888	1.979^*^	45.505	11.939^***^	55.897^*^
	(1.50)	(−0.16)	(1.51)	(1.67)	(1.40)	(2.85)	(1.72)
Sobel test		Mediator variable: Peer context 0.045^***^ Indirect association—Positive conduction		Mediator variable: Positive peers 1.586^***^ Indirect association—Positive conduction		Mediator variable: Negative peers −0.607^***^ Indirect association—Negative conduction	
Ind_eff Test (P-val)		0.013 Indirect effect established		0.001 Indirect effect established		0.039 Indirect effect established	
Controls	Yes	Yes	Yes	Yes	Yes	Yes	Yes
City fixed effects	Yes	Yes	Yes	Yes	Yes	Yes	Yes
School fixed effects	Yes	Yes	Yes	Yes	Yes	Yes	Yes
Observations (*N*)	6,713	6,713	6,713	6,713	6,713	6,713	6,713
Adjusted *R^2^*	0.104	0.067	0.105	0.104	0.107	0.124	0.109

^***^, ^**^, and ^*^ indicate statistical significance at the 1%, 5%, and 10% levels, respectively.

This study further divides peer context into positive peer context and negative peer context. The results show that physical exercise duration is significantly associated with both positive and negative peer contexts. After incorporating the key independent variable and the mediating variables, the coefficients for positive peer context is significantly positive, while those for negative peer context is significantly negative. This indicates that physical exercise may be indirectly associated with better mental health through positive peer context, while its association with negative peer context may be linked to poorer mental health. Specifically, positive peer context associated with longer physical exercise duration is linked to better adolescent mental health, whereas negative peer context is linked to poorer mental health. Detailed results are presented in Columns (4) to (7) of Table 7.

In summary, physical exercise may be linked to better mental health through more favorable peer context. However, when students are exposed to peers exhibiting negative behaviors during physical activities, such peer context may be associated with poorer mental health.

### 4.5 Heterogeneity analysis

#### 4.5.1 School sports facilities

The availability of school sports facilities may shape students' opportunities for regular physical exercise. Facilities such as swimming pools, sports fields, and gymnasiums provide organized and accessible spaces for physical education classes and extracurricular activities, thereby creating a more supportive environment for physical activity. In this study, school sports facilities are measured according to whether a school has at least one of the following facilities: a swimming pool, sports field, or gymnasium. Schools equipped with at least one of these facilities are categorized as having sports facilities, while schools without any of these facilities are categorized as lacking sports facilities.

To examine whether the association between physical exercise and mental health differs across school facility conditions, this study conducts subgroup regressions using all available matched survey observations. The results are reported in Columns (1) and (2) of Table 8, with the corresponding sample sizes reported in the table. The results show that, among students attending schools with sports facilities, physical exercise is positively and significantly associated with mental health. By contrast, among students attending schools without sports facilities, the coefficient of physical exercise is not statistically significant. This pattern indicates that the positive association between physical exercise and mental health is more evident in schools where sports facilities are available. The result is consistent with the view that school-based physical activity resources may influence the extent to which students can translate exercise participation into mental health benefits.

**Table 8:** Heterogeneity test.

Variables	(1)	(2)	(3)	(4)
	No sports facilities	With sports facilities	High economic level	Low economic level
Average physical exercise time	−0.557	0.283^**^	0.305^**^	−0.012
	(−0.59)	(2.25)	(2.28)	(−0.03)
Physical exercise	−7.160^**^	−1.853^***^	−2.058^***^	−1.458
	(−2.38)	(−4.66)	(−4.84)	(−1.32)
Gender	0.033	0.749	0.716	−0.390
	(0.01)	(1.56)	(1.42)	(−0.25)
Only child	0.417	−0.250	−0.154	−0.714
	(0.18)	(−0.79)	(−0.47)	(−0.59)
Household registration type	1.399	−0.622	−0.777	1.053
	(0.33)	(−0.78)	(−0.88)	(0.61)
Physical health	2.633	3.383^***^	3.467^***^	2.390^***^
	(1.66)	(15.66)	(14.71)	(4.47)
Parental divorce	0.664	0.014	−1.204	3.378^*^
	(0.14)	(0.02)	(−1.31)	(1.87)
Relationship with parents	3.058	1.809^**^	2.135^***^	1.261
	(0.72)	(2.52)	(2.60)	(0.87)
Parental education level	0.776	0.547^***^	0.524^***^	0.343
	(0.60)	(3.62)	(3.29)	(0.71)
Parental occupation	5.708	0.663	0.667	0.289
	(1.19)	(1.10)	(1.08)	(0.10)
Teacher-student relationship	5.756^**^	2.730^***^	3.014^***^	2.339^***^
	(2.34)	(7.68)	(7.80)	(2.67)
School ranking	12.176^*^	1.462	0.777	35.499
	(1.66)	(0.26)	(0.13)	(1.26)
School type	0.000	−2.880	1.117	−65.227^**^
	(.)	(−0.52)	(0.19)	(−2.33)
Non-sports facilities at school	11.503^*^	0.346	−5.441	158.530^**^
	(1.98)	(0.02)	(−0.38)	(2.26)
Constant	4.526	48.397	56.049^*^	−213.734
	(0.17)	(1.49)	(1.69)	(−1.31)
City fixed effects	Yes	Yes	Yes	Yes
School fixed effects	Yes	Yes	Yes	Yes
Observations (*N*)	130	6,583	5,737	976
Adjusted *R^2^*	0.120	0.101	0.100	0.078

^***^, ^**^, and ^*^ indicate statistical significance at the 1%, 5%, and 10% levels, respectively.

#### 4.5.2 Household economic status

This study further examines whether the association between physical exercise and mental health differs across household economic conditions. Household economic status is measured using parents' self-reported household economic condition. Families reporting “very difficult” or “fairly difficult” economic conditions are classified as the low household economic status group, while families reporting “average,” “fairly wealthy,” or “very wealthy” conditions are classified as the non-low household economic status group.

Subgroup regressions are conducted using all available matched survey observations, and the results are shown in Columns (3) and (4) of Table 8. Among students from non-low household economic status families, physical exercise is positively and significantly associated with mental health. In contrast, among students from low household economic status families, the coefficient of physical exercise is not statistically significant. This result suggests that the positive association between physical exercise and mental health is more pronounced among students from families with relatively better economic conditions. One possible explanation is that household economic resources may affect the quality, continuity, and context of adolescents' physical activity participation, including access to exercise equipment, extracurricular sports opportunities, and parental support for regular physical activity. Therefore, the heterogeneity results suggest that family resource conditions may shape the observed relationship between physical exercise and students' mental health.

## 5 Discussion

This study provides large-sample observational evidence on the positive association between physical exercise and the mental health of middle school students in China. Students who reported longer exercise duration tended to report better mental health, and this association remained stable across several empirical specifications. Rather than viewing physical exercise as an isolated behavioral factor, the findings can be understood within a broader movement-behavior framework in which physical activity, sedentary behavior, cognitive functioning, and psychological adjustment are interrelated aspects of adolescent development. Existing research suggests that physical activity may be linked to youth mental health through neurobiological, psychosocial, and behavioral processes, including improved emotional regulation, social interaction, cognitive control, and school adaptation (Lubans et al., 2016). From this perspective, evidence on 24-h movement behaviors and device-based physical activity further indicates that adolescents' daily activity patterns are closely connected with psychological functioning, cognitive difficulties, and later mental health outcomes (Gao et al., 2024; Werneck et al., 2025). Therefore, the positive association observed in this study should be interpreted not simply as a direct exercise–mental health link, but as part of a broader developmental process through which movement behaviors may relate to adolescents' psychological and school functioning.

The role of academic stress can also be interpreted within this integrated framework. In the school context, academic stress represents a key psychosocial condition shaping adolescents' emotional burden and mental health. The mediation results show that longer exercise duration is associated with lower academic stress, and lower academic stress is associated with better mental health. This pattern is consistent with the idea that physical activity may be related to mental health partly through its connection with academic functioning and executive processes. Prior studies on movement-behavior guidelines, academic-related outcomes, and executive functioning suggest that physical activity may support learning-related adjustment by improving attention regulation, inhibitory control, and other cognitive resources that are relevant to school performance and stress management (Bao et al., 2024; Zhang et al., 2025b, 2026). Accordingly, academic stress in this study should be understood as a school-specific psychosocial factor linking physical exercise, academic adaptation, and mental health. However, because the variables are measured contemporaneously, this pathway should be interpreted as an associated psychosocial channel rather than definitive evidence of temporal mediation.

Peer context provides another important perspective for interpreting the association between physical exercise and mental health. School-based physical exercise often takes place in group settings, where students interact with classmates through cooperation, competition, and informal communication. The results suggest that positive peer context is associated with better mental health, whereas negative peer context is associated with poorer mental health. This finding is consistent with research emphasizing the social and emotional functions of physical activity participation among adolescents. For example, Lubans et al. (2012) showed that physical activity programs may be related to social and emotional wellbeing among at-risk youth, while studies on adolescent sport and peer interaction also suggest that physical activity settings can provide opportunities for social support, belonging, and interpersonal adjustment. At the same time, the findings indicate that peer-related contexts are not uniformly beneficial. Physical activity settings may create opportunities for friendship formation and group belonging, but they may also expose some students to negative peer behaviors or exclusion. Therefore, school-based physical activity should be organized in ways that strengthen supportive peer interaction and reduce negative peer experiences.

The heterogeneity results show that the positive association between physical exercise and mental health is more evident among students attending schools with sports facilities and those from non-low household economic status families. This finding suggests that the mental health relevance of physical exercise may depend on the resources and contexts in which exercise takes place. Students with better school facilities may have more stable access to organized, safe, and diverse physical activity opportunities. Similarly, students from families with relatively better economic conditions may receive more support for regular exercise participation, including access to equipment, extracurricular sports opportunities, and parental encouragement. In contrast, students from economically disadvantaged families or schools with limited sports facilities may face greater constraints in transforming exercise participation into psychological benefits. Thus, the heterogeneity results highlight the importance of considering family and school resources when designing physical activity interventions for adolescent mental health.

Taken together, the findings suggest that physical exercise is associated with adolescent mental health through a combination of academic, social, and contextual factors. Rather than viewing exercise as an isolated individual behavior, it may be more appropriate to understand it as embedded in students' school routines, peer environments, and family resource conditions. For educational practice, this implies that promoting adolescent mental health through physical activity requires more than increasing exercise duration alone. Schools should also improve the quality and accessibility of physical activity environments, support positive peer interaction, and pay particular attention to students from economically disadvantaged families or schools with limited sports resources. At the same time, given the observational and cross-sectional nature of the data, the findings should be interpreted as associations rather than causal effects. Future research using longitudinal or experimental designs is needed to further examine the temporal ordering and causal pathways among physical exercise, academic stress, peer context, and mental health.

## 6 Conclusions and recommendations

### 6.1 Research conclusions

Based on the CEPS data, this study uses fixed-effects models to examine the association between physical exercise and the mental health of middle school students. Robustness checks and instrumental-variable analysis are further conducted to assess the stability of the results. The main conclusions are as follows: (1) Physical exercise duration is positively associated with students' mental health, and students reporting longer exercise duration tend to have higher mental health scores; (2) Academic stress and peer context appear to be potential psychosocial pathways linking physical exercise and mental health. Specifically, longer exercise duration is associated with lower academic stress and more favorable peer context, while negative peer context is associated with poorer mental health; (3) The positive association between physical exercise and mental health is more pronounced among students from non-low household economic status families and those attending schools with sports facilities. These findings provide useful evidence for the implementation of the “Healthy China 2030” strategy, particularly in promoting school-based physical activity and adolescent mental health support. Given the observational and cross-sectional nature of the data, these conclusions should be interpreted as associations rather than causal effects.

### 6.2 Recommendations

According to the research findings, the study proposes the following policy recommendations. At the school level, increased investment in sports facilities should be prioritized, along with the promotion of team sports activities to foster positive interaction among students. Additionally, curriculum design should be optimized to ensure that students have sufficient daily time for physical exercise. At the family level, parents should be encouraged to view physical exercise as a crucial component of their children's overall wellbeing and to actively participate in physical activities with their children to promote both physical and mental health. At the public policy level, public investment in community and school-based sports resources should be increased, particularly in under-resourced areas, alongside the introduction of subsidized programs for low-income families to ensure equitable access to physical exercise for all students. These recommendations aim to create more supportive conditions for students' physical activity participation and psychological wellbeing through the integration of resources from schools, families, and society, and provide policy support for the implementation of the “Healthy China 2030” strategy.

## Data Availability

The original contributions presented in the study are included in the article/supplementary material, further inquiries can be directed to the corresponding author.
